# DNA Polymerase ζ-Dependent Lesion Bypass in *Saccharomyces cerevisiae* Is Accompanied by Error-Prone Copying of Long Stretches of Adjacent DNA

**DOI:** 10.1371/journal.pgen.1005110

**Published:** 2015-03-31

**Authors:** Olga V. Kochenova, Danielle L. Daee, Tony M. Mertz, Polina V. Shcherbakova

**Affiliations:** Eppley Institute for Research in Cancer and Allied Diseases, University of Nebraska Medical Center, Omaha, Nebraska, United States of America; Duke University, UNITED STATES

## Abstract

Translesion synthesis (TLS) helps cells to accomplish chromosomal replication in the presence of unrepaired DNA lesions. In eukaryotes, the bypass of most lesions involves a nucleotide insertion opposite the lesion by either a replicative or a specialized DNA polymerase, followed by extension of the resulting distorted primer terminus by DNA polymerase ζ (Polζ). The subsequent events leading to disengagement of the error-prone Polζ from the primer terminus and its replacement with an accurate replicative DNA polymerase remain largely unknown. As a first step toward understanding these events, we aimed to determine the length of DNA stretches synthesized in an error-prone manner during the Polζ-dependent lesion bypass. We developed new *in vivo* assays to identify the products of mutagenic TLS through a plasmid-borne tetrahydrofuran lesion and a UV-induced chromosomal lesion. We then surveyed the region downstream of the lesion site (in respect to the direction of TLS) for the presence of mutations indicative of an error-prone polymerase activity. The bypass of both lesions was associated with an approximately 300,000-fold increase in the mutation rate in the adjacent DNA segment, in comparison to the mutation rate during normal replication. The hypermutated tract extended 200 bp from the lesion in the plasmid-based assay and as far as 1 kb from the lesion in the chromosome-based assay. The mutation rate in this region was similar to the rate of errors produced by purified Polζ during copying of undamaged DNA in vitro. Further, no mutations downstream of the lesion were observed in rare TLS products recovered from Polζ-deficient cells. This led us to conclude that error-prone Polζ synthesis continues for several hundred nucleotides after the lesion bypass is completed. These results provide insight into the late steps of TLS and show that error-prone TLS tracts span a substantially larger region than previously appreciated.

## Author Summary

Genomic instability is associated with multiple genetic diseases. Endogenous and exogenous DNA-damaging factors constitute a major source of genomic instability. Mutations occur when DNA lesions are bypassed by specialized translesion synthesis (TLS) DNA polymerases that are less accurate than the normal replicative polymerases. The discovery of the remarkable infidelity of the TLS enzymes at the turn of the century immediately suggested that their contribution to replication must be tightly restricted to sites of DNA damage to avoid excessive mutagenesis. The actual extent of error-prone synthesis that accompanies TLS *in vivo* has never been estimated. We describe a novel genetic approach to measure the length of DNA synthesized by TLS polymerases upon their recruitment to sites of DNA damage. We show that stretches of error-prone synthesis associated with the bypass of a single damaged nucleotide span at least 200 and sometimes up to 1,000 nucleotide-long segments, resulting in more than a 300,000-fold increase in mutagenesis in the surrounding region. We speculate that processive synthesis of long DNA stretches by error-prone polymerases could contribute to clustered mutagenesis, a phenomenon that allows for rapid genome changes without significant loss of fitness and plays an important role in tumorigenesis, the immune response and adaptation.

## Introduction

Genomic stability is continuously threatened by endogenous and exogenous DNA-damaging factors. Unrepaired lesions stall the replication machinery, because the highly selective active sites of replicative DNA polymerases cannot accept abnormally shaped nucleotides [1, 2]. The bypass of replication impediments is facilitated by specialized translesion synthesis (TLS) polymerases. In humans, these include the Y-family enzymes Polη, Polι, Polκ, and Rev1, and the B-family enzyme Polζ. The yeast *Saccharomyces cerevisiae* has homologs of Polη, Rev1 and Polζ [3]. A more open active site allows the TLS polymerases to accommodate a variety of DNA lesions and catalyze synthesis on damaged templates [4, 5]. While important for tolerating DNA damage, TLS is a highly mutagenic process because of the miscoding potential of the damaged nucleotides and the inherently lower fidelity of the specialized polymerases. It is a major source of environmentally induced mutations and a significant contributor to spontaneous mutagenesis. Particularly, yeast and mammalian cells lacking Polζ or its partner Rev1 are completely deficient in mutagenesis induced by most DNA-damaging agents [3, 6, 7].

TLS is a two-step process that involves insertion of a nucleotide opposite the lesion and extension of the resulting distorted primer terminus. The insertion can be performed by a replicative polymerase or one of the TLS polymerases, depending on the type of lesion. With the exception of *cis-syn* cyclobutane pyrimidine dimers, where Polη is also able to facilitate the extension step, extension of the aberrant primer terminus is usually catalyzed by Polζ [8–10]. This unique role of Polζ underlies its absolute requirement for damage-induced mutagenesis: the extension of primer terminus containing a wrong nucleotide is essential for conversion of the misincorporation into a permanent change in the DNA. While the molecular details of the insertion and extension steps have been studied extensively for a variety of lesions, the subsequent processes leading to the replacement of the TLS polymerases with accurate replicative enzymes are poorly understood. The low fidelity of the TLS polymerases suggests that their contribution to DNA synthesis past the lesion site must be tightly regulated. The coordination of TLS with the ongoing DNA replication has been considered in the context of the following two models [6, 11]. In the model referred to as “polymerase switching” or “TLS at the fork”, the stalled replicative polymerase hands the primer terminus over to a TLS polymerase to allow for the bypass to occur and then returns to resume high-fidelity replication. In this scenario, TLS operates at the fork and provides for continuous synthesis of the daughter strand. In contrast, in the “gap-filling” model, stalling of the replicative polymerase is followed by a quick re-priming of replication downstream of the blocking lesion, which leaves a gap between the site of the lesion and the site of the restart. TLS polymerases then act post-replicatively to facilitate filling of these gaps. The possibility of direct switching from a replicative to a TLS polymerase and back is in excellent agreement with the biochemical properties of these enzymes and their behavior on templates containing a single replication-blocking lesion *in vitro* [12, 13]. A bulk of evidence, however, suggests that TLS *in vivo* might predominantly occur as postreplicative gap filling (discussed thoroughly in [6, 11, 14]). Perhaps the strongest arguments are the lack of a noticeable decrease in the rate of fork progression in TLS-deficient mutants [15–17] and the accumulation of discontinuities in the newly synthesized DNA observed in multiple studies of UV-irradiated *E*. *coli*, yeast and mammalian cells [17–22]. The possibility of TLS polymerases acting in a gap-filling mode is illustrated by the participation of human Polκ in DNA synthesis during nucleotide excision repair (NER) [23] and by a recent study suggesting that Polζ is required for filling of lesion-containing, NER-generated gaps in non-dividing yeast cells [24]. Electron microscopy showed that gaps of up to 1,000 nucleotides are left behind the forks in replicating UV-treated yeast [17]. Nonetheless, all of the existing evidence for each of the TLS models is indirect. Whether TLS polymerases actually operate in these gaps, which undoubtedly exist, and what proportion of TLS events occur at the fork rather than postreplicatively, remains to be established.

The mode of TLS could be an important factor that determines the extent of synthesis performed by the error-prone TLS polymerases. In the case of direct polymerase exchange at the fork, it would seem rational for the replicative polymerase to return to the primer terminus as soon as its activity is no longer impeded by the lesion. *In vitro*, the eukaryotic replicative polymerases Polδ and Polε can resume processive DNA synthesis once a TLS polymerase elongates the primer by two to five nucleotides past the lesion [13]. It has been argued that the stretches of TLS synthesis should be just sufficiently long to prevent degradation of the TLS product by the proofreading activity of a replicative polymerase and not much longer to limit the accumulation of mutations in the downstream region [12]. Replicative DNA polymerases can sense distortions in the duplex DNA within 3–6 base pairs from the primer terminus [12, 13, 25]. Therefore, extending the error-prone synthesis further than a few nucleotides past the lesion would provide little benefit for the efficiency of TLS while delaying the fork progression and increasing the mutation load. In contrast, in the gap-filling model of TLS, the progression of replication does not require switching from a TLS to a replicative polymerase once the lesion is bypassed. A replicative polymerase may not be readily available outside of the replication fork, and the TLS polymerases could be well positioned to fill a large portion of the gap or even close it completely. A possibility of error-prone TLS proceeding well beyond the lesion and generating additional “untargeted”, or “hitchhiking”, mutations in the adjacent region has been considered previously [26, 27] but was never thoroughly investigated. In eukaryotes, the involvement of Polζ in the extension step during the bypass of most lesions makes this polymerase a likely candidate for continuing the synthesis past the lesion. The fidelity of purified Polζ *in vitro* was estimated to be 5.6 x 10^-4^ mutations per nucleotide synthesized [28]. While this is dramatically lower than the fidelity of the replicative enzymes, Polζ is still the most accurate among the eukaryotic TLS polymerases. It could be the best choice for synthesis of extended DNA stretches if it were to be done by a TLS polymerase.

In this study, we aimed to determine how much DNA is synthesized in an error-prone manner after Polζ completes the bypass of a plasmid-borne tetrahydrofuran lesion (THF) or a UV-induced chromosomal lesion *in vivo*. We reasoned that if Polζ-dependent TLS is accompanied by extensive copying of the adjacent undamaged DNA, these regions should exhibit an increased frequency of mutation. We developed new genetic assays that allowed us to identify and isolate the products of Polζ-dependent TLS occurring *in vivo* and examine the region surrounding the lesion site for the presence of mutations characteristic of a TLS polymerase activity. The results argue that Polζ copies several hundred nucleotides of DNA past the lesion after completing the bypass, which leads to a 300,000-fold increase in the mutation rate in this region. This demonstrates that TLS in eukaryotic cells is associated with mutagenesis not only at the lesion site, but in the extended adjacent area as well. In addition, these findings provide support for the previously hypothetical role of Polζ in filling the daughter strand gaps formed opposite lesions in replicating DNA.

## Results

### A Genetic System to Identify the Products of Mutagenic THF Bypass

**Fig 1:**
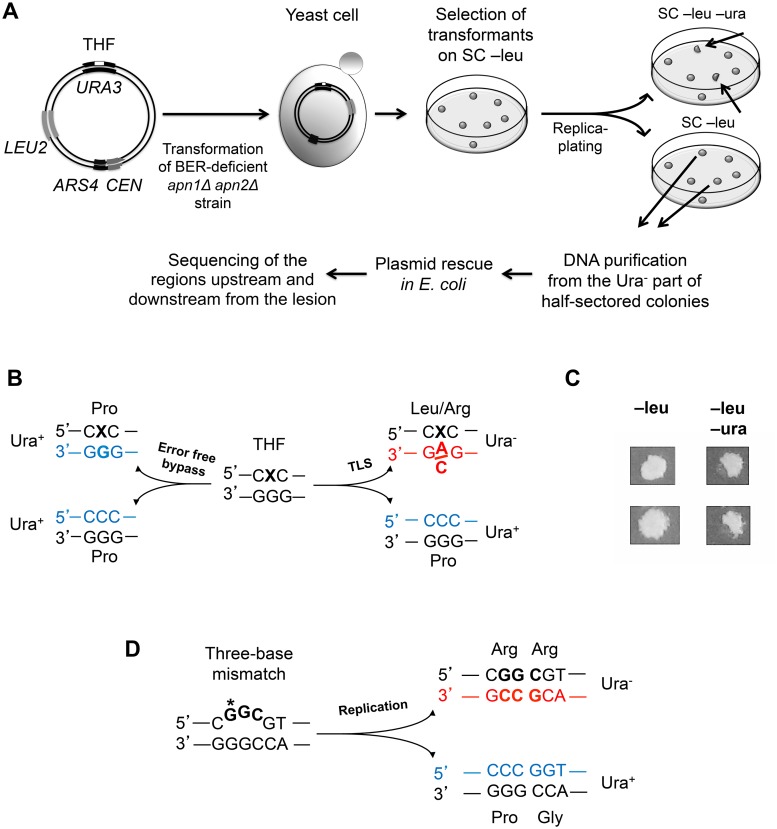
A genetic system to analyze the products of TLS through a site-specific THF lesion. (A) Sequential steps of the THF bypass assay. A double-stranded centromeric (*CEN*) plasmid containing a THF lesion at position 605 of the *URA3* gene (white rectangle), a selectable marker (*LEU2*) and the yeast replication origin (*ARS4*) is introduced into the *apn1Δ apn2Δ* yeast strain. Transformants are selected for leucine prototrophy and then replica-plated on the medium lacking uracil to identify half-sectored colonies (shown by the arrows). The Ura^-^ parts of the half-sectored colonies originate from cells that underwent error-prone TLS through the THF (see text for details). (B) Possible phenotypic outcomes of the THF bypass at position 605. The lesion is indicated with an “X”. The newly synthesized strands are in blue (correct incorporation) or in red (incorrect incorporation), with nucleotides across from the lesions highlighted in bold. The amino acids at the corresponding position of the protein and the resulting phenotypes (Ura^+^ or Ura^-^) are listed next to the triplet sequences. (C) Identification of TLS events by the half-sectored colony phenotype. Images of two colonies exhibiting full-growth on medium lacking leucine (SC—leu) and half-growth on medium lacking uracil (SC—leu–ura) are shown. (D) A schematic showing the “bubble”-type mismatch in the control plasmid and phenotypes associated with copying of each strand. Nucleotides that differ from the wild-type *URA3* sequence are in bold. The position 605 of the *URA3* gene is marked with an asterisk. Other symbols are as in (B).

This study aimed to determine whether Polζ-dependent TLS *in vivo* is associated with low-fidelity DNA synthesis past the lesion, and if so, define the size of DNA region copied in an error-prone manner. We first developed a system for the analysis of TLS through THF, an abasic site analog. Mutagenic bypass of both natural and artificial abasic sites in yeast requires Polζ for the extension step [29, 30] and is, thus, a good model of Polζ-dependent TLS. We have constructed a double-stranded plasmid containing a THF lesion at a specific position in the *URA3* gene, a yeast replication origin *ARS4*, and the *LEU2* gene for selection of cells containing the plasmid (Fig. 1A). Replication of this plasmid was studied in *apn1Δ apn2Δ* strains lacking the two yeast apurinic/apyrimidinic endonucleases to prevent removal of the lesion by the base excision repair system. Replication of the damaged strand in the yeast cells could be accomplished through several pathways, including TLS, error-free bypass utilizing an undamaged homologous sequence as a template, or a recombination-dependent mechanism. To distinguish the products of Polζ-dependent TLS from other events, we took advantage of the earlier observations that TLS through THF predominantly involves an A or C incorporation across from the lesion [31–33]. The THF was designed to replace a C in the wild-type *URA3* sequence, such that an A or C insertion opposite it would produce a Ura^-^ phenotype. Accurate bypass utilizing an undamaged template or repair of the lesion prior to replication (presumably infrequent in the *apn1Δ apn2Δ* strains but possible, Ref. [34]) would produce a DNA strand with a G at this position and a Ura^+^ phenotype (Fig. 1B). TLS events, thus, result in the formation of half-sectored colonies, where the Ura^-^ and Ura^+^ halves result from the copying of the THF-containing and the complementary undamaged strands, respectively (Fig. 1C). Non-TLS events result in the formation of Ura^+^ colonies. According to the previous reports, a small percentage of TLS cases involve a T or G insertion opposite the THF (e.g. 1% and 8% of all TLS events, respectively, during replication of a double-stranded THF-containing plasmid; Ref. [31]). The T insertion results in a Ura^-^ phenotype in our system and, therefore, is identified as a TLS event. The G insertion preserves the wild-type *URA3* sequence and cannot be distinguished from non-TLS events. Accordingly, TLS products containing a G across from the THF position were not included in the analysis described below. The half-sectored colony phenotype was observed in ~1% of the transformants with the THF-containing plasmids (a total of 394 sectored colonies among ~40,000 transformants analyzed). While this is consistent with the previous observations that the great majority of THF bypass events occurs through mechanisms other than TLS [31], non-sectored colonies in our system could also result from replication fork uncoupling and copying of the undamaged strand [17]. To detect TLS-associated mutations, we isolated the plasmids from the Ura^-^ part of the half-sectored colonies and analyzed a 1.7-kb region beginning at the original lesion site and extending in the direction of TLS, as well as a 550-bp region upstream of the lesion site in respect to the direction of TLS, by Sanger sequencing. The sequence corresponding to the oligonucleotide used to construct the THF-containing plasmid (20 nt before and 26 nt after the lesion) was excluded from the analysis, because mutations in this region could result from errors during the *in vitro* synthesis of the oligonucleotide. All of the plasmids analyzed (a total of 394) contained a base substitution at the THF position, confirming the mutagenic TLS event. The distance from the lesion site at which additional mutations were found served as an estimate of the length of the error-prone TLS tracts in this system.

Because of the accumulation of random damage in the ssDNA used for the plasmid construction, replication of the THF-containing plasmid is expected to result in a high rate of mutations in the vector sequence not related to the THF bypass. To evaluate the contribution of these “background” mutations to the overall mutagenesis in the 1.7-kb region downstream of the THF site, we designed a control plasmid constructed via the same procedure as the THF-containing plasmid but with no deliberately introduced lesion. To be able to analyze the progeny of the same strand that is replicated via error-prone TLS in the THF bypass assay, we engineered the two strands of the control plasmid to confer different phenotypes. One strand contains the wild-type *URA3* sequence, while the other contains a three-nucleotide substitution (the *ura3-103*,*104* mutation) at the position equivalent to that of the THF lesion, resulting in a three-base mismatch in the plasmid (Fig. 1D). Approximately 20% of transformants with this plasmid produced half-sectored colonies consistent with the replication of the plasmid and segregation of the *URA3* and *ura3-103*,*104* alleles into the daughter cells. The proportion of half-sectored colonies only mildly increased in mismatch repair (MMR)-deficient *msh2Δ* strains (from 21% to 33% on average), indicating that the “bubble”-type mismatch is inefficiently corrected by MMR. The non-sectored colonies could possibly result from the presence of more than one plasmid copy in some cells, which could preclude segregation of the Ura^-^ phenotype, loss of a fraction of daughter plasmids and/or repair of the heteroduplex by an unknown mechanism distinct from MMR. We did not attempt to distinguish between these possibilities and used only half-sectored colonies for the analysis of control replication products. All of the plasmids isolated from the Ura^-^ part of the half-sectored colonies contained the *ura3-103*,*104* allele sequence at the site of the of the original mismatch, as expected from accurate copying of the Ura^-^ strand. DNA sequence analysis of the surrounding region provided an estimate of the frequency of mutations associated with our method of plasmid construction.

### The Length of DNA Fragments Synthesized in an Error-Prone Manner during THF Bypass

**Fig 2:**
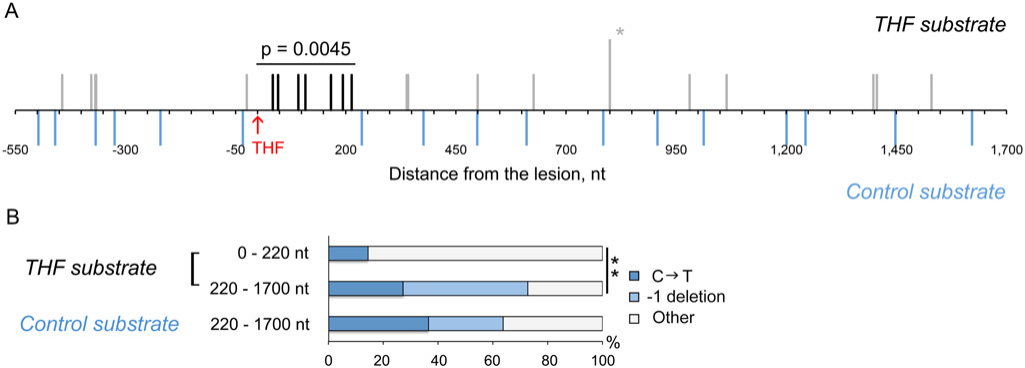
THF bypass is associated with increased mutagenesis downstream of the lesion. (A) Distribution of mutations found in the products of TLS through the THF lesion. The THF position is indicated in red. Each vertical line represents a single mutation; the mutation found twice is marked with the asterisk. Mutations in the TLS products within 220 bp from the lesion are in black, other mutations in TLS products are in grey. Blue lines below the horizontal scale bar represent mutations found in the control substrates without the THF. The data are based on DNA sequence analysis of 394 THF bypass products and 456 products of the control plasmid replication. P-value (Fisher’s exact test) indicates the significance of differences in the frequency of mutation in the 220-nucleotide region between TLS products and control plasmids. (B) Types of mutations observed in the THF bypass products and control plasmids. C → T changes are shown for the transcribed strand that is exposed as ssDNA during the plasmid construction. The double asterisk indicates a statistically significant difference (p = 0.0347, Fisher’s exact test).

A total of 394 THF bypass products and 456 products of the control plasmid replication were analyzed by DNA sequencing. As expected, the majority of TLS events resulted in an A (243/394; 62%) or C (80/394; 20%) incorporation opposite the lesion. T incorporation occurred in 18% of all cases (71/394). A total of 18 mutations were found in the downstream region at distances between 34 and 1529 nucleotides from the THF (Fig. 2A; Table 1). These “hitchhiking” mutations were noticeably concentrated within an approximately 220-nucleotide segment immediately adjacent to the lesion. Although 11 mutations were found among the 456 control plasmids downstream of the lesion site, their distribution was significantly different from that in the TLS products. Mutations in the control plasmids were randomly distributed throughout the sequenced region, with none of the 11 mutations occurring within the first 220 nucleotides, in contrast to ~40% in the THF bypass products (p = 0.0045, Fisher’s exact test). The rate of mutation in the 220-nucleotide region next to the THF site constituted 8.1 x 10^-5^ per nucleotide (Table 2). This exceeds the genome-wide mutation rate in yeast by approximately 300,000-fold and is close to the rate of errors reported for copying of undamaged DNA by purified Polζ *in vitro* (5.6 x 10^-4^, [28]). The frequency of mutations immediately upstream of the lesion site did not differ from that in the control plasmids (Fig. 2A), consistent with the idea that the patch of increased mutagenesis resulted from error-prone DNA synthesis initiated at the lesion site. The frequency of mutations downstream of the lesion was reduced to the background level as the distance from the lesion exceeded 220 nucleotides. Accordingly, the types of mutations in these distant regions were very similar to those in the control plasmids (predominantly C→T transitions and -1 deletions). In contrast, only one C→T transition and no -1 frameshifts were found in the 220-bp region adjacent to the lesion site (Fig. 2B, Table 1). We, therefore, concluded that mutations present in the TLS products outside of the 220-bp region must have resulted from damage of ssDNA during the plasmid construction, and only those observed within the 220-bp region are indicative of error-prone DNA synthesis associated with the THF bypass. We also sequenced the 220-bp region in 47 THF bypass products and 57 control plasmids recovered from *msh2Δ* strains to determine whether errors made during TLS-associated synthesis are corrected by MMR. We observed no increase in the frequency of untargeted mutations over that in MMR-proficient strains (Table 2), indicating that MMR does not operate in TLS tracts. This is in agreement with a previous report that MMR does not efficiently correct errors made by Polζ [35]. Taken together, these observations suggest that the error-prone synthesis typically continues for approximately 200 nucleotides after the THF bypass is completed. Because of the high level of background mutagenesis in this system, we cannot exclude a possibility that a minor fraction of TLS events could involve more extensive low-fidelity synthesis.

**Table 1:** Mutations found in THF bypass products and in the control plasmids.

Substrate	Nucleotide inserted opposite the THF	Additional mutations downstream of the THF position			
		Type of mutation^a^	Distance from the lesion site (nt)	Position in the *URA3* gene^b^	Position in the vector^c^
THF	C	A → G	- 444		4516
	A	C → T	- 378		4450
	A	G → C	- 368		4440
	A	C ins	- 367		4439
	A	TC → AA	- 24	629	4096
	C	T → A	+ 34	571	4038
	T	A → C	+ 46	559	4030
	A	A → C	+ 92	513	3984
	A	GAT del	+ 108	497	3968
	A	A → C	+ 168	437	3908
	C	C → T	+ 193	412	3883
	A	G → T	+ 213	392	3863
	A	G del	+ 338	267	3738
	T	G del	+ 341	264	3735
	T	A del	+ 498	107	3578
	C	G → C	+ 626		3450
	A	A del	+ 799		3277
	T	A del	+ 799		3277
	C	C → T	+ 980		3096
	C	G → C	+ 1064		3021
	A	A → T	+ 1397		2688
	T	C → T	+ 1405		2680
	A	C → T	+ 1529		2556
Control	NA	A del	- 498		4575
	NA	C → T	- 460		4537
	NA	C ins	- 368		4445
	NA	T del	- 325		4402
	NA	C → G	- 221	826	4298
	NA	C → T	- 34	639	4111
	NA	A → G	+ 236	369	3840
	NA	G del	+ 376	229	3700
	NA	A del	+ 498	107	3578
	NA	T → C	+ 610		3466
	NA	T del	+ 784		3292
	NA	C → T	+ 907		3178
	NA	C → T	+ 1012		3073
	NA	G → T	+ 1209		2876
	NA	C → T	+ 1242		2843
	NA	A → C	+ 1447		2638
	NA	C → T	+ 1621		2812

^a^Nucleotide changes in the strand complementary to the THF-containing strand are shown.

^b^Numbering for the *URA3* gene is from the first nucleotide of the open reading frame.

^c^Numbering for the vector if from the first nucleotide following the *ARS4* sequence.

nt, nucleotide; del, deletion; ins, insertion; NA, not applicable.

**Table 2:** Rate of mutation in various genomic regions in cells undergoing error-prone TLS.

Region	Mutation rate per nucleotide			
	In MMR^+^ strains		In MMR^-^ strains	
	THF bypass-associated	UV lesion bypass-associated	THF bypass-associated	UV lesion bypass-associated
Near the lesion^a^	8.1 x 10^-5^	6.7 x 10^-5^	< 9.7 x 10^-5^	< 7.7 x 10^-5^
Genome-wide	2.2 x 10^-10^ ^b^	0.4 x 10^-5^ ^c^	ND	ND

The rate of mutation downstream of the lesion site in the THF and UV lesion bypass experiments was calculated as follows: μ = m/(L*n), where *m* is the number of mutations, *L* is the length of the DNA region analyzed by sequencing (in nucleotides), and *n* is the number of TLS products examined. For UV lesion bypass experiment, the background mutation rate (0.6 x 10^-5^; calculated from sequencing of the 1-kb region next to the *ura3-G764A* site in UV-induced Can^r^ mutants) was subtracted for the observed rate of TLS-associated mutation. The rate of TLS-associated mutation in MMR-deficient strains was estimated similarly taking into account the rate of background mutation in the corresponding control experiments.

^a^Within 220 bp from the lesion for the THF bypass and within 1000 bp for the UV lesion bypass.

^b^Spontaneous genome-wide mutation rate as calculated in [78]

^c^Genome-wide mutation rate in cells undergoing UV-induced mutagenesis was estimated based on the observance of three mutations within the sequenced 5-kb region in the UV-induced Can^r^ mutants.

ND, not determined.

### A Genetic System to Identify the Products of TLS through a UV-Induced Chromosomal Lesion

**Fig 3:**
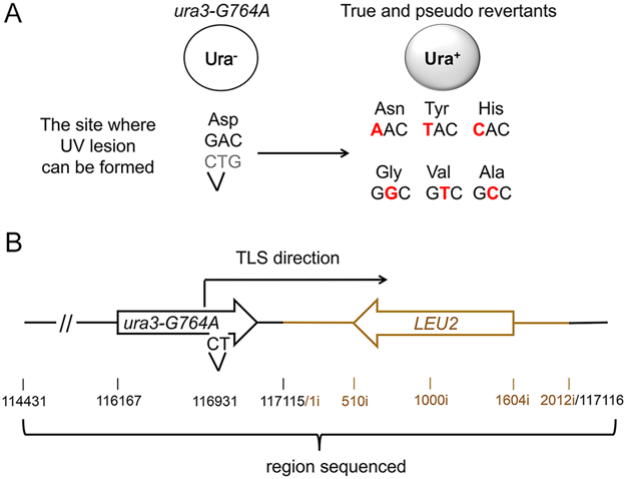
A genetic system to analyze the products of TLS through a chromosomal UV lesion. (A) The *ura3-G764A* allele and possible UV-induced single nucleotide substitutions that lead to the Ura^+^ phenotype (marked in red). The sequence of the non-transcribed DNA strand is in black, and the transcribed strand is in grey. The site of potential UV lesion formation is indicated with a “V”. (B) A schematic showing the structure of the *ura3-G764A-LEU2* cassette in chromosome *V*, the direction of the UV lesion bypass, and the region that was analyzed by DNA sequencing. The 2-kb *Hpa*I *LEU2* fragment used as a selectable marker for introducing the *ura3-G764A* allele into the chromosome is in dark yellow. Open arrows indicate open reading frames. Black numbers show chromosomal nucleotide position in respect to the left telomere; dark yellow numbers with the “i” index show nucleotide position within the *LEU2* insert in respect to the end of the *Hpa*I fragment.

**Fig 4:**
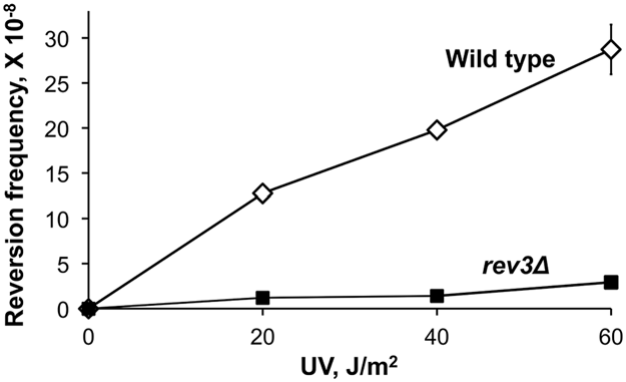
Frequency of UV-induced reversion of the *ura3-G764A* allele in the wild-type and Polζ-deficient (*rev3Δ*) strains. The data are mean frequencies for at least six determinations. Error bars are shown unless they are smaller than the plot symbol and represent standard errors.

As described previously, sensitivity of the plasmid TLS assay is limited by the high background likely resulting from spontaneous damage during the plasmid construction. To overcome this limitation and to ascertain that the extended stretches of error-prone DNA synthesis is not a peculiar feature of the plasmid assay, we next developed an approach to study mutagenesis associated with the Polζ-dependent bypass of a chromosomal DNA lesion. Because creating a unique site-specific abasic site in a yeast chromosome is technically challenging, we chose to use a lesion induced by UV irradiation at a specific dipyrimidine sequence in the chromosomal *URA3* gene. *Cis-syn* cyclobutane pyrimidine dimers and (6–4) photoproducts are major types of DNA lesion induced by UV irradiation and can be generated at any of the four pyrimidine doublets, TT, CT, TC, and CC [36]. Error-prone bypass of UV lesions *in vivo*, like that of abasic sites, occurs via a Polζ/Rev1-dependent pathway [37–39]. We introduced a single nucleotide substitution at position 764 of the chromosomal *URA3* gene (the *ura3-G764A* mutation) that leads to a Ura^-^ phenotype and creates a dipyrimidine sequence (TC), a possible site for UV lesion formation (Fig. 3). The *ura3-G764A* strains can revert to the Ura^+^ phenotype via several base substitutions at the 3' C or 5' T of the dinucleotide (Fig. 3A). The occurrence of either substitution upon UV irradiation of yeast cells indicates that the lesion formation and the mutagenic TLS have taken place at this site. The frequency of the *ura3-G764A* reversion increased in a dose-dependent manner in wild-type strains, but not in *rev3Δ* mutants lacking Polζ (Fig. 4). This indicated that UV irradiation readily induces lesions that are bypassed via Polζ-dependent synthesis to produce the Ura^+^ revertants. DNA sequence analysis of the *URA3* locus of 165 independent revertants obtained after irradiation with 60 J/m^2^ UV light showed that all of the revertants contained base substitutions at 3' C, 5' T or both positions of the dipyrimidine at the site of *ura3-G764A* mutation (S1 Table). Thus, the system is highly efficient in the identification of products of mutagenic TLS through a site-specific chromosomal lesion. To determine the extent of error-prone synthesis associated with the bypass of this lesion, total DNA was isolated from the 165 Ura^+^ revertants, and 2.5-kb regions upstream and downstream from the reversion site in respect to the direction of TLS were amplified by PCR and sequenced (Fig. 3B).

### The Length of DNA Fragments Synthesized in an Error-Prone Manner during the Bypass of a Chromosomal UV Lesion

**Fig 5:**
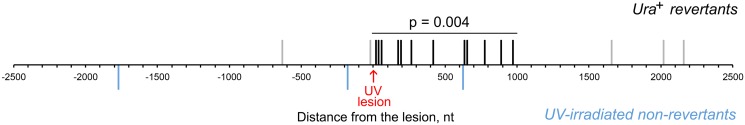
UV lesion bypass is associated with increased mutagenesis downstream of the lesion. The position of the presumed UV lesion at the *ura3-G764A* mutation site is indicated in red. Distribution of untargeted mutations in UV-induced Ura^+^ revertants is shown above the horizontal scale bar. Each vertical line represents a single mutation. Mutations found within 1000 bp downstream from the lesion are in black, those in other regions are in grey. Blue lines below the horizontal scale bar represent mutations found in UV-induced Can^r^ mutants of the *ura3-G764A* strain. The data are based on DNA sequence analysis of 165 independent Ura^+^ revertants and 161 independent Can^r^ mutant. P-value (Fisher’s exact test) indicates the significance of differences in the frequency of mutation in the 1-kb region between Ura^+^ revertants and Can^r^ controls.

Similar to the THF bypass, TLS through the chromosomal UV lesion was frequently accompanied by additional mutations in the 2.5-kb region downstream of the lesion site. In 12 cases of reversion at the *ura3-G764A* site, a mutation at the 5’ or 3’ nucleotide of the TC doublet was associated with a mutation at the next G presumably not involved in the lesion formation (+1 position; S1 Table). Because the accuracy of nucleotide incorporation at this position is likely severely affected by the distorted DNA structure at the damaged site, we did not include these mutations in the calculation of mutation rate in the adjacent region. A total of 15 additional mutations were found in the 165 TLS products at distances between 16 to 2155 nucleotides downstream of the reversion site (Fig. 5; Table 3). As in the case of the THF bypass, the untargeted mutations were noticeably concentrated in the region immediately adjacent to the lesion, but the hypermutated stretch now extended as far as 1000 nucleotides from the presumed lesion position (Fig. 5). The mutation rate in this 1000-bp segment constituted 6.7 x 10^-5^ per nucleotide (Table 2), which is similar to the level of mutagenesis we observed in the products of the THF bypass. To confirm that these mutations are associated with TLS at the site of *ura3-G764A* mutation, we repeated the experiment but selected for cells that underwent mutation at the *CAN1* locus rather than the Ura^+^ reversion. The *CAN1* is located ~83 kb away from the *ura3-G764A* allele in the same chromosome *V*. We detected only one DNA sequence change in the 1-kb region downstream of the *ura3-G764A* mutation site among 161 independent UV-induced Can^r^ mutant (Fig. 5). This indicated that the untargeted mutations in the Ura^+^ revertants were, indeed, related to the TLS through a nearby lesion and did not simply reflect a high level of mutagenesis in the genome of irradiated cells. The frequency of mutations upstream of the *ura3-G764A* site was low and similar to that in the Can^r^ controls, consistent with the idea that the error-prone synthesis initiated at the site of *ura3-G764A* mutation. The rare mutations we observed in the Can^r^ controls and in the Ura^+^ revertants outside the hypermutated 1-kb region likely resulted from additional UV-induced lesions. According to previous estimates, the dose of 60 J/m^2^ used in our experiments is expected to induce approximately one lesion per 1–2 kb [40, 41]. The frequency of mutation we observed in the Can^r^ controls (which is indicative of the average frequency of mutagenic lesions in the genome of cells undergoing UV-induced mutation) is about 100-fold lower. This is consistent with the notion that the majority of lesions are repaired in NER-proficient cells, and only a fraction of the remaining lesions are mutagenic.

**Table 3:** Mutations found in UV-induced Ura^+^ revertants of the *ura3-G764A* strain and Can^r^ controls.

	Nucleotide change at positions 763–765 of the *URA3* gene^a^	Additional mutations downstream of the presumed UV lesion		
		Mutation type^a^	Distance from the lesion site (nt)	Chromosomal position^b^
Ura^+^ revertants(TLS at the site of *ura3-G764A* mutation)	GAC→G**C**C	A → T	- 635	116294
	GAC→G**T**C	G → A	- 23	116906
	GAC→**A**AC	G → A	+ 16	116946
	GAC→**A**AC	A → G	+ 34	116964
	GAC→**A**AC	A → G	+ 40	116970
	GAC→**A**AC	A ins	+ 177	117107
	GAC→**A**AC	C ins	+ 190	5i
	GAC→G**G**C	A ins	+ 262	77i
	GAC→**A**A**T**	T ins	+ 414	229i
	GAC→G**G**C	A → T	+ 631	446i
	GAC→**C**AC	A ins	+ 636	451i
	GAC→**A**AC	C → T	+ 772	587i
	GAC→G**TT**	G → A	+ 886	701i
	GAC→**A**AC	G → A	+ 968	783i
	GAC→G**T**C	G → A	+ 1654	1469i
	GAC→**A**AC	G → T	+ 2015	1830i
	GAC→G**C**C	C → T	+ 2155	1970i
Can^r^ controls (no TLS at the site of *ura3-G764A* mutation)	NA	A → T	- 1774	115155
	NA	T → C	- 181	116748
	NA	C → T	+ 621	436i

^a^Nucleotide changes (bold) are shown for the coding DNA strand complementary to the strand containing the dipyrimidine sequence at positions 763–764.

^b^Numbers with the “i” index indicate the position of the mutation in the inserted *LEU2* gene fragment (see Fig. 3 for a more detailed explanation of the numbering system). Abbreviations are as in Table 1.

Polζ is required for the extension step during the mutagenic bypass of THF and UV-induced lesions [29, 33, 38, 39] and could, thus, be responsible for the error-prone synthesis in the downstream region. To exclude the possibility that the observed high rate of mutations resulted from synthesis by the other low-fidelity yeast polymerase, Polη, we sequenced the 1-kb region downstream of the *ura3-G764A* mutation site in 165 Ura^+^ revertants obtained in the *rad30Δ* background (S2 Table) after exposure to the same dose of UV irradiation (60 J/m^2^). The reversion frequency was not significantly affected by the inactivation of *RAD30*. Five mutations were found at distances of 505–1026 nucleotides from the reversion site, which corresponds to a mutation rate of 3 x 10^-5^. This is similar to what we observed in the *RAD30* ^*+*^ strain and argues against a major role of Polη in mutagenesis downstream of the lesion site. In contrast, Polζ appeared to be required for the generation of untargeted mutations. Although very little induced mutagenesis could be seen in *rev3Δ* strains lacking Polζ (Fig. 4), the frequency of Ura^+^ revertants at the dose of 60 J/m^2^ exceeded the spontaneous reversion frequency approximately three-fold, so the rare revertants resulting from Polζ-independent bypass could be recovered. Sequencing of the 1-kb region downstream of the *ura3-G764A* mutation site in 231 Ura^+^ revertant obtained in the *rev3Δ* background (S3 Table) detected no untargeted mutations. This indicated that the long stretches of hypermutation downstream of the lesion are specifically associated with Polζ-dependent TLS and strongly implicate Polζ in the generation of these mutations. Similar to the THF bypass experiments, sequencing of the 1-kb region from UV-induced Ura^+^ revertants obtained in the *msh2Δ* background showed that the frequency of untargeted mutations was not increased in the absence of MMR (Table 2), confirming that MMR does not correct errors in TLS tracts.

## Discussion

Previous studies of the Polζ-dependent TLS focused primarily on events at the lesion site [8, 29, 31, 42–47]. These studies have established Polζ as a key player in the extension of distorted primer termini resulting from nucleotide insertion opposite lesions by other polymerases. This function underlies the renowned requirement of Polζ for mutagenesis at the damage location. In the present work, we used novel genetic assays where the products of TLS can be identified phenotypically to demonstrate that the bypass of the THF and the UV-induced lesions is associated with a dramatic increase in mutagenesis in the adjacent region. The following arguments suggest that this mutagenesis likely results from continuous synthesis of long DNA stretches by Polζ. First, the essential role of Polζ in the extension step of the bypass puts this polymerase in a perfect position to carry on synthesis beyond the lesion. Second, the mutation rate in the region downstream of the lesion (~10^-4^ per bp) is comparable to the rate of errors observed during Polζ-dependent copying of undamaged DNA *in vitro* (5.6 x 10^-4^ per bp; Ref. [28]). Third, the untargeted mutations disappear in Polζ-deficient *rev3Δ* strains. Fourth, the level of untargeted mutagenesis remained high in *rad30* mutants lacking Polη, the only other TLS polymerase in yeast capable of copying complex templates. We can also exclude a reduction in the fidelity of replicative polymerases as the cause of TLS-associated mutagenesis, because this would be expected to result in a genome-wide elevation of the mutation rate, which is not observed.

The rate and specificity of mutation downstream of the lesions argues that it results from the copying of adjacent undamaged DNA by Polζ and not from its recruitment to sites of secondary lesions in single-stranded regions formed after the replication arrest. The frequency of mutation triggered by the formation of ssDNA alone, without additional mutagenic treatment, is at least an order of magnitude lower than that observed in our experiments [48]. The ssDNA-mediated mutagenesis is also characterized by the abundance of C→T changes in the exposed strand (~43% of all base substitutions; Ref. [48]), which are completely absent in the 200-bp segment adjacent to the THF site in our study (Table 1). While the UV lesion bypass products contained C→T transitions (Table 3), the mutation rate downstream of the lesion site still greatly exceeded that expected from spontaneous damage in persistent ssDNA stretches (Table 2 and Ref. [48]). Therefore, we conclude that the distribution of mutations in the vicinity of the site-specific lesions (Figs. 2A and 5) directly reflects the extent of continuous synthesis by Polζ. Based on this, we estimate that, *in vivo*, Polζ copies at least 200 and sometimes up to 1,000 nucleotides of undamaged template upon completing the lesion bypass. This nicely parallels previous electron microscopy studies showing that the single-stranded gaps left behind the replication forks in UV-irradiated yeast are typically smaller than 400 nucleotides, but longer gaps (more than 1000 nucleotides) could be seen in a fraction of replication intermediates [17]. If TLS, as it is currently viewed, occurs predominantly in these gaps, Polζ must be filling a substantial portion of the gaps. Further studies would be needed to determine whether Polζ is solely responsible for the gap filling or if it is later replaced by a replicative polymerase such as Pol δ. The TLS assays we developed can be used as a tool to address this question if mapping of TLS stretches is complemented by accurate measuring of ssDNA regions accumulating next to the lesions.

We observed a notable difference in the distribution of mutations in the THF *versus* the UV lesion bypass assay, which might reflect a previously unappreciated aspect of TLS regulation. Mutations associated with the THF bypass concentrated within 220 nucleotides from the lesion (Fig. 2; Table 1). In contrast, mutations that accompanied the UV lesion bypass were distributed nearly randomly across the adjacent 1-kb region, becoming less frequent only beyond that point (Fig. 5). This indicates that the bypass of the chromosomal UV lesion is associated with longer stretches of error-prone synthesis. It seems likely that the extent of Polζ-dependent synthesis may be limited by the length of the single-stranded region remaining after re-priming of replication downstream of the lesion. This length could vary depending on the lesion position in the leading or lagging strand template. Stalled lagging strand synthesis is likely restarted with the initiation of the next Okazaki fragment, so the size of single-stranded gaps on the lagging strand would not exceed the size of Okazaki fragments (140–175 nucleotides according to the recent estimates, Refs. [49, 50]). However, replication restart on the leading strand requires additional regulatory mechanisms, and the re-priming might occur at a greater distance from the lesion. Although the direction of replication through the *URA3* gene in our THF bypass assay is unknown, we believe the lesion is likely to be encountered by the lagging strand machinery in the majority of cases. The THF lesion is located at comparable distances from the centromere-proximal and centromere-distal sides of the replication origin *ARS4* in the pRS315-*URA3* OR2 plasmid (Fig. 1A). Because of the inhibitory effect of the repetitive centromeric sequences on the fork progression [51], the lesion-containing region is likely to be first approached by the fork coming from the centromere-distal side of *ARS4*. This would put the THF lesion in the lagging strand template. In contrast, the UV-induced lesion in chromosome *V* is likely located in the leading strand template. The end of the *URA3* gene in a genetically unmanipulated chromosome *V* corresponds to the beginning of the replication termination zone (the region between 117 and 123 kb in Fig. 3B; Refs. [52, 53]). The *LEU2* insertion (Fig. 3B) presumably moves the termination zone away from the *URA3*, placing the site of lesion formation in the leading strand template at a distance of at least 1 kb from the termination zone. The lesion position in the opposite DNA strands in the THF and the UV lesion bypass assays could potentially explain the differences in the length of DNA fragments synthesized in an error-prone manner. While this explanation at present remains hypothetical, the assays we described here could easily be adapted in the future to explore the role of replication fork dynamics and asymmetry in regulating the extent of Polζ-dependent synthesis.

The findings described here bring a new twist to understanding the consequences of DNA damage. The extent of error-prone synthesis downstream of the lesions is such that, regardless of whether the lesion occurs in a functionally important position, the probability of inactivating a nearby gene is extremely high. We estimate that, with ~7% of TLS tracts containing an additional mutation beyond the lesion site (this study), and ~1/3 of all base substitutions and the majority of frameshifts in coding regions affecting the gene function [54], a TLS tract spanning a coding region will destroy the gene in ~4% of cases. While targeted mutations are undoubtedly the primary cause of damage-induced mutagenesis, given the high proportion of essential genes (e. g. 1/3 of all genes in yeast), the untargeted mutations contribute to DNA damage sensitivity and pose an upper limit to the absolute number of unrepaired lesions that can be tolerated by a cell. Extended tracks of error prone synthesis can also lead to the accumulation of multiple mutations in a localized area without causing hypermutability across the genome. The localized hypermutability provides a mechanism for rapid genome changes while minimally affecting fitness and is believed to play an important role in several biological processes, including tumorigenesis, immune response and adaptation (discussed in [55–60]). While a major cause of clustered mutations was suggested to be the enzymatic deamination of cytosines in ssDNA, processive synthesis of long stretches of DNA by error-prone polymerases could conceivably contribute to this phenomenon as well. The two processes, in fact, are interrelated: the excision of uracil resulting from cytosine deamination by uracil DNA glycosylases produces abasic sites [61]. Subsequently, the deaminase-induced mutagenesis is, in part, mediated by Polζ-dependent TLS [57]. The long stretches of error-prone synthesis are also likely relevant to other situations where mutagenic processes promote adaptation, evolution or human disease and where the role of clustered mutations is yet to be established. For example, Polζ/Rev1-dependent TLS is believed to be responsible for the acquired drug resistance and the development of secondary tumors in patients undergoing chemotherapy with DNA-damaging agents [62–65]. The ability of TLS enzymes to generate multiple mutations in extended stretches of DNA likely accelerates the emergence of chemoresistance. Future molecular characterization of therapy-resistant tumors could help clarify the role of the TLS-associated localized hypermutability in tumor evolution.

## Materials and Methods

### Strains and Plasmids

The haploid *Saccharomyces cerevisiae* strains PS1001/PS1002 (*MATα ade5 lys2-Tn5-13 trp1-289 his7-2 leu2-3*,*112 ura3Δ apn1Δ*::*loxP apn2Δ*::*loxP)* and OK29/30 (*MATα ade5-1 lys2*::*InsE*_*A14*_ *trp1–289 his7-2 leu2-3*,*112 ura3-G764A-LEU2*) were used in the THF and UV lesion bypass assays, respectively. PS1001 and PS1002 are two independent isolates of the same genotype derived from CG379Δ [66, 67] by disruption of the *APN1* and *APN2* genes by the *loxP-LEU2-loxP* and *loxP-kanMX-loxP* cassettes, respectively. The cassettes were PCR-amplified from pUG73 [68] and pUG6 [69], and the disruption was followed by the Cre/*loxP*-mediated marker removal [69]. OK29 and OK30 are two independent isolates of the same genotype engineered as follows using E134 (same as OK29/30, but *ura3-52*; [70]) as the starting material. First, a Ura^+^ derivative of E134 (named E134^+^) was obtained by M. R. Northam in our laboratory by transformation with a PCR fragment containing the wild-type *URA3* gene. The *ura3-G764A* mutation was created by site-directed mutagenesis in a yeast integrative vector containing the *URA3* and *LEU2* genes cloned into pUC18 [71], yielding pUC18-*ura3-G764A*-or1. OK29 and OK30 were then constructed by replacing the wild-type chromosomal *URA3* gene of E134^+^ with the *ura3-G764A-LEU2* cassette amplified by PCR from pUC18-*ura3-G764A*-or1. The primers for amplification had 20 bp of homology to pUC18 regions flanking the cassette at the 3’ end and 45 bp of homology to the chromosome *V* sequences upstream and downstream of the *URA3* gene at the 5’ end. The *LEU2* insertion next to the *ura3-G764A* in OK29/30 does not affect the function of the *URA3* gene or the Ura^-^ phenotype conferred by the mutation. The *rev3Δ*, *rad30Δ* and *msh2Δ* mutants of OK29/30 and *msh2Δ* mutants of PS1001/1002 were constructed by transformation with PCR-generated DNA fragments carrying the *kanMX* cassette flanked by short sequence homology to *REV3*, *RAD30* or *MSH2*.

The pRS315-*URA3* OR2 plasmid [72] containing the *URA3* gene cloned into the *Hin*dIII site of pRS315 [73] was kindly provided by Dr. Youri Pavlov (University of Nebraska Medical Center, Omaha, U.S.A). In addition to the *URA3*, it carries the *LEU2* selectable marker, the yeast autonomous replicative sequence *ARS4*, a yeast centromere sequence, and the f1 phage origin of replication. The single-stranded DNA (ssDNA) form of pRS315-*URA3* OR2 contains the transcribed *URA3* strand. *Escherichia coli* F’ strain DH12S (Invitrogen) and M13KO7 helper phage (New England Biolabs) were used for isolation of the pRS315-*URA3* OR2 ssDNA. The *E*. *coli* strains XL10-Blue and MC1061 (Invitrogen) were used for plasmid rescue from yeast cells and for propagation of plasmid DNA.

### Construction of the Double-Stranded Plasmid with a Site-Specific THF Lesion

The single-stranded pRS315-*URA3* OR2 phagemid was purified as described in [74] with some modifications. The DH12S strain transformed with the phagemid was grown in LB medium to an optical density at 600 nm of 0.05 and then infected with M13KO7 at a final concentration of 1 x 10^8^ pfu/ml. On the following day, the bacteriophage particles were precipitated from the culture supernatant by stirring in 4% polyethelene glycol—0.5 M NaCl at 4°C for 1 h and subsequent centrifugation at 4,000 x g for 30 min. The pellets were washed with 10 mM Tris-HCl and resuspended in 10 mM Tris-HCl pH 8.0. The cell debris was then removed by centrifugation at 60,000 x g for 15 min at 4°C. To pellet the phage particles, a subsequent overnight centrifugation was performed under the same conditions. The pelleted bacteriophage particles were resuspended in 10 mM TE buffer. To remove residual fragments of bacterial DNA or RNA that could anneal to the pRS315-*URA3* OR2 ssDNA, the bacteriophage particles were treated with 120 U/ml T4 DNA polymerase (New England Biolabs) and 5 μg/ml RNAse A (USB) in NEB2 buffer (New England Biolabs) at 37°C for 2 h. The treatment was done in the absence of dNTPs to utilize the 3’-exonuclease rather than the DNA polymerase activity of T4 DNA polymerase. The reaction was stopped by incubation with 5 μg/ml Proteinase K (Sigma-Aldrich) at 55°C for 30 min. The pRS315-*URA3* OR2 ssDNA was then purified from pre-cooled bacteriophage particles by three sequential extractions with phenol, two extractions with phenol-chloroform, and one extraction with chloroform, followed by ethanol precipitation. Samples were shaken gently to prevent shearing of the DNA. Purified ssDNA was stored in 10 mM TE buffer at -80°C.

The double-stranded plasmid containing a site-specific THF lesion and a control undamaged plasmid were constructed by annealing oligonucleotides 5’-AGGTTACGATTGGTTGATTATGACACXCGGTGTGGGTTTAGATGACA-3’ (Oligos etc), where “X” is THF, and 5’-AGGTTACGATTGGTTGATTATGACACGGCGTGTGGGTTTAGATGACA-3’ (IDT), respectively, to the pRS315-*URA3* OR2 ssDNA and synthesizing the remainder of the second strand by T7 DNA polymerase. The oligonucleotides are complementary to the *URA3* nucleotides 579–625. The control oligonucleotide contains three bases (underlined) that do not match the wild-type *URA3* sequence and produce a triple CCG → GGC substitution (the *ura3-103*,*104* allele) resulting in a Ura^-^ phenotype. The oligonucleotides were PAGE-purified and annealed to the pRS315-*URA3* OR2 ssDNA by incubating a two-fold molar excess of the oligonucleotide with the 400 ng of ssDNA at 72°C for 2 min in T4 DNA ligase buffer (New England Biolabs) and then cooling slowly to room temperature. The whole volume of the annealing mix was then incubated with 10 U of T7 DNA polymerase, 200 μM dNTPs, 4 mM ATP and 10 U of T4 DNA ligase (New England Biolabs) in T7 DNA polymerase buffer at 37°C for 1.5 h. The reactions were then treated with Proteinase K (Invitrogen) at 37°C for 20 min. The covalently closed double-stranded plasmids were isolated from 0.8% agarose gel by centrifugation through premade Sephadex-10 columns (Pharmacia Fine Chemicals) as previously described [75].

### Isolation and Analysis of the THF Bypass Products

Double-stranded THF-containing and control plasmids were introduced into the yeast cells by polyethylene-glycol-mediated transformation [76]. The strains were grown at 30°C in rich liquid YPDAU medium [77] prior to transformation. Transformants were selected on synthetic complete medium without leucine (SC—leu), and three-day-old colonies were replica-plated on synthetic complete medium without leucine and uracil (SC −leu—ura) to score half-sectored phenotype. Total yeast DNA was purified from the Ura^-^ part of the half-sectored colonies using the MasterPure Yeast DNA Purification Kit (Epicentre). To isolate plasmids from the total DNA samples, 5–7 μl of each sample was used for transformation of the XL10-Blue or MC1061 *E*.*coli* strain, and plasmid DNA was purified from individual bacterial colonies by using the High-Speed Plasmid Mini Kit (IBI Scientific). A portion of the plasmid comprising 550 nucleotides upstream and 1.7 kb downstream of the THF position (in respect to the direction of the presumed TLS), as well as the corresponding region in the progeny of the control plasmid, was analyzed by DNA sequencing.

### Measurement of the Mutation Frequency in UV-Irradiated Cells

To measure the frequency of UV-induced *ura3-G764A* reversion, appropriately diluted overnight cultures of the *ura3-G764A* mutants were plated on synthetic complete medium and SC—ura medium and irradiated immediately with 254 nm UV light at doses indicated in Fig. 4. The plates were incubated for five days at 30°C. Mutant frequencies were then calculated as the ratio of the number of revertants on selective plates to the number of colonies on synthetic complete plates multiplied by the dilution factor.

### Isolation and Analysis of UV Lesion Bypass Products

To isolate UV-induced Ura^+^ revertants or canavanine-resistant (Can^r^) mutants of the OK29 and OK30 strains or their *rev3Δ*, *rad30Δ* or *msh2Δ* derivatives, the strains were streaked for single colonies on YPDAU plates and grown for three days at 30°C. Liquid cultures were then started in YPDAU from the individual colonies and grown to the stationary phase. A total of 200 μl of two-fold concentrated saturated cultures were spread on a SC—ura plate or SC—arg supplemented with 60 μg/ml L-canavanine, irradiated immediately with 60 J/m^2^ of 254 nm UV light, and incubated for seven days (for Ura^+^ revertants) or five days (for Can^r^ mutants) to allow for colony formation. One revertant or Can^r^ mutant was randomly picked from each plate for DNA sequence analysis. Total yeast DNA was isolated from the revertants and Can^r^ mutants using the MasterPure Yeast DNA Purification Kit (Epicentre). A 5 kb-region comprising 2.5 kb upstream and 2.5 kb downstream of the *ura3-G764A* mutation site was amplified by PCR using Pfu DNA polymerase kindly provided by Dr. Farid Kadyrov (Southern Illinois University School of Medicine, Carbondale, U.S.A.) and sequenced.

## Supporting Information

S1 Table Nucleotide changes at the site of the presumed UV lesion at positions 763–765 of the *URA3* gene in UV-induced revertants of the *ura3-G764A* strain. The location of the potential photolesion site and the true and pseudo reversion pathways are explained in detail in Fig. 3.
(DOCX)

S2 Table Nucleotide changes at the site of the presumed UV lesion at positions 763–765 of the *URA3* gene in UV-induced revertants in the *rad30Δ* background. The location of the potential photolesion site and the true and pseudo reversion pathways are explained in detail in Fig. 3.
(DOCX)

S3 Table Nucleotide changes at the site of the presumed UV lesion at positions 763–765 of the *URA3* gene in UV-induced revertants in the *rev3Δ* background. The location of the potential photolesion site and the true and pseudo reversion pathways are explained in detail in Fig. 3.
(DOCX)

## References

[1] McCulloch SD, Kunkel TA. The fidelity of DNA synthesis by eukaryotic replicative and translesion synthesis polymerases. Cell Res. 2008;18: 148–161. 10.1038/cr.2008.4 18166979 PMC3639319

[2] Broyde S, Wang L, Rechkoblit O, Geacintov NE, Patel DJ. Lesion processing: high-fidelity versus lesion-bypass DNA polymerases. Trends Biochem Sci. 2008;33: 209–219. 10.1016/j.tibs.2008.02.004 18407502 PMC2717799

[3] Boiteux S, Jinks-Robertson S. DNA repair mechanisms and the bypass of DNA damage in *Saccharomyces cerevisiae* . Genetics. 2013;193: 1025–1064. 10.1534/genetics.112.145219 23547164 PMC3606085

[4] Yang W. An Overview of Y-Family DNA Polymerases and a Case Study of Human DNA Polymerase η. Biochemistry. 2014;53: 2793–2803. 10.1021/bi500019s 24716551 PMC4018060

[5] Sale JE, Lehmann AR, Woodgate R. Y-family DNA polymerases and their role in tolerance of cellular DNA damage. Nat Rev Mol Cell Biol. 2012;13: 141–152. 10.1038/nrm3289 22358330 PMC3630503

[6] Waters LS, Minesinger BK, Wiltrout ME, D'Souza S, Woodruff RV, Walker GC. Eukaryotic translesion polymerases and their roles and regulation in DNA damage tolerance. Microbiol Mol Biol Rev. 2009;73: 134–154. 10.1128/MMBR.00034-08 19258535 PMC2650891

[7] Lange SS, Takata K, Wood RD. DNA polymerases and cancer. Nat Rev Cancer. 2011;11: 96–110. 10.1038/nrc2998 21258395 PMC3739438

[8] Lawrence CW. Cellular functions of DNA polymerase ζ and Rev1 protein. Adv Protein Chem. 2004;69: 167–203. 15588843 10.1016/S0065-3233(04)69006-1

[9] Prakash S, Johnson RE, Prakash L. Eukaryotic translesion synthesis DNA polymerases: specificity of structure and function. Annu Rev Biochem. 2005;74: 317–353. 15952890 10.1146/annurev.biochem.74.082803.133250

[10] Livneh Z, Ziv O, Shachar S. Multiple two-polymerase mechanisms in mammalian translesion DNA synthesis. Cell Cycle. 2010;9: 729–735. 20139724 10.4161/cc.9.4.10727

[11] Lehmann AR, Fuchs RP. Gaps and forks in DNA replication: Rediscovering old models. DNA Repair (Amst). 2006;5: 1495–1498. 16956796 10.1016/j.dnarep.2006.07.002

[12] Fujii S, Fuchs RP. Defining the position of the switches between replicative and bypass DNA polymerases. EMBO J. 2004;23: 4342–4352. 15470496 10.1038/sj.emboj.7600438 PMC524402

[13] McCulloch SD, Kokoska RJ, Chilkova O, Welch CM, Johansson E, Burgers PM, et al Enzymatic switching for efficient and accurate translesion DNA replication. Nucleic Acids Res. 2004;32: 4665–4675. 15333698 10.1093/nar/gkh777 PMC516052

[14] Ulrich HD. Timing and spacing of ubiquitin-dependent DNA damage bypass. FEBS Lett. 2011;585: 2861–2867. 10.1016/j.febslet.2011.05.028 21605556

[15] Elvers I, Johansson F, Groth P, Erixon K, Helleday T. UV stalled replication forks restart by re-priming in human fibroblasts. Nucleic Acids Res. 2011;39: 7049–7057. 10.1093/nar/gkr420 21646340 PMC3167624

[16] Callegari AJ, Clark E, Pneuman A, Kelly TJ. Postreplication gaps at UV lesions are signals for checkpoint activation. Proc Natl Acad Sci USA. 2010;107: 8219–8224. 10.1073/pnas.1003449107 20404181 PMC2889594

[17] Lopes M, Foiani M, Sogo JM. Multiple mechanisms control chromosome integrity after replication fork uncoupling and restart at irreparable UV lesions. Mol Cell. 2006;21: 15–27. 16387650 10.1016/j.molcel.2005.11.015

[18] Rupp WD, Howard-Flanders P. Discontinuities in the DNA synthesized in an excision-defective strain of *Escherichia coli* following ultraviolet irradiation. J Mol Biol. 1968;31: 291–304. 4865486 10.1016/0022-2836(68)90445-2

[19] Iyer VN, Rupp WD. Usefulness of benzoylated naphthoylated DEAE-cellulose to distinguish and fractionate double-stranded DNA bearing different extents of single-stranded regions. Biochim Biophys Acta. 1971;228: 117–126. 4926026 10.1016/0005-2787(71)90551-x

[20] Prakash L. Characterization of postreplication repair in *Saccharomyces cerevisiae* and effects of *rad6*, *rad18*, *rev3* and *rad52* mutations. Mol Gen Genet. 1981;184: 471–478. 7038396 10.1007/BF00352525

[21] Lehmann AR. Postreplication repair of DNA in ultraviolet-irradiated mammalian cells. J Mol Biol. 1972;66: 319–337. 5037019 10.1016/0022-2836(72)90418-4

[22] Meneghini R. Gaps in DNA synthesized by ultraviolet light-irradiated WI38 human cells. Biochim Biophys Acta. 1976;425: 419–427. 130925 10.1016/0005-2787(76)90006-x

[23] Ogi T, Limsirichaikul S, Overmeer RM, Volker M, Takenaka K, Cloney R, et al Three DNA polymerases, recruited by different mechanisms, carry out NER repair synthesis in human cells. Mol Cell. 2010;37: 714–727. 10.1016/j.molcel.2010.02.009 20227374

[24] Kozmin SG, Jinks-Robertson S. The mechanism of nucleotide excision repair-mediated UV-induced mutagenesis in nonproliferating cells. Genetics. 2013;193: 803–817. 10.1534/genetics.112.147421 23307894 PMC3583999

[25] Zhang Y, Wu X, Guo D, Rechkoblit O, Geacintov NE, Wang Z. Two-step error-prone bypass of the (+)- and (-)-*trans*-*anti*-BPDE-*N* ^2^-dG adducts by human DNA polymerases η and κ. Mutat Res. 2002;510: 23–35. 12459440 10.1016/s0027-5107(02)00249-x

[26] Ruiz-Rubio M, Bridges BA. Mutagenic DNA repair in *Escherichia coli*. XIV. Influence of two DNA polymerase III mutator alleles on spontaneous and UV mutagenesis. Mol Gen Genet. 1987;208: 542–548. 3312950 10.1007/BF00328153

[27] Maor-Shoshani A, Reuven NB, Tomer G, Livneh Z. Highly mutagenic replication by DNA polymerase V (UmuC) provides a mechanistic basis for SOS untargeted mutagenesis. Proc Natl Acad Sci USA. 2000;97: 565–570. 10639119 10.1073/pnas.97.2.565 PMC15370

[28] Zhong X, Garg P, Stith CM, Nick McElhinny SA, Kissling GE, Burgers PM, et al The fidelity of DNA synthesis by yeast DNA polymerase ζ alone and with accessory proteins. Nucleic Acids Res. 2006;34: 4731–4742. 16971464 10.1093/nar/gkl465 PMC1635245

[29] Haracska L, Unk I, Johnson RE, Johansson E, Burgers PM, Prakash S, et al Roles of yeast DNA polymerases δ and ζ and of Rev1 in the bypass of abasic sites. Genes Dev. 2001;15: 945–954. 11316789 10.1101/gad.882301 PMC312678

[30] Gibbs PE, Lawrence CW. Novel mutagenic properties of abasic sites in *Saccharomyces cerevisiae* . J Mol Biol. 1995;251: 229–236. 7643399 10.1006/jmbi.1995.0430

[31] Pages V, Johnson RE, Prakash L, Prakash S. Mutational specificity and genetic control of replicative bypass of an abasic site in yeast. Proc Natl Acad Sci USA. 2008;105: 1170–1175. 10.1073/pnas.0711227105 18202176 PMC2234110

[32] Kow YW, Bao G, Minesinger B, Jinks-Robertson S, Siede W, Jiang YL, et al Mutagenic effects of abasic and oxidized abasic lesions in *Saccharomyces cerevisiae* . Nucleic Acids Res. 2005;33: 6196–6202. 16257982 10.1093/nar/gki926 PMC1275587

[33] Zhao B, Xie Z, Shen H, Wang Z. Role of DNA polymerase η in the bypass of abasic sites in yeast cells. Nucleic Acids Res. 2004;32: 3984–3994. 15284331 10.1093/nar/gkh710 PMC506798

[34] Torres-Ramos CA, Johnson RE, Prakash L, Prakash S. Evidence for the involvement of nucleotide excision repair in the removal of abasic sites in yeast. Mol Cell Biol. 2000;20: 3522–3528. 10779341 10.1128/mcb.20.10.3522-3528.2000 PMC85644

[35] Lehner K, Jinks-Robertson S. The mismatch repair system promotes DNA polymerase ζ-dependent translesion synthesis in yeast. Proc Natl Acad Sci USA. 2009;106: 5749–5754. 10.1073/pnas.0812715106 19307574 PMC2667058

[36] Ravanat JL, Douki T, Cadet J. Direct and indirect effects of UV radiation on DNA and its components. J Photochem Photobiol B. 2001;63: 88–102. 11684456 10.1016/s1011-1344(01)00206-8

[37] Gibbs PE, McDonald J, Woodgate R, Lawrence CW. The relative roles in vivo of *Saccharomyces cerevisiae* Pol η, Pol ζ, Rev1 protein and Pol32 in the bypass and mutation induction of an abasic site, T-T (6–4) photoadduct and T-T *cis-syn* cyclobutane dimer. Genetics. 2005;169: 575–582. 15520252 10.1534/genetics.104.034611 PMC1449107

[38] Kozmin SG, Pavlov YI, Kunkel TA, Sage E. Roles of *Saccharomyces cerevisiae* DNA polymerases Polη and Polζ in response to irradiation by simulated sunlight. Nucleic Acids Res. 2003;31: 4541–4552. 12888515 10.1093/nar/gkg489 PMC169879

[39] Yu SL, Johnson RE, Prakash S, Prakash L. Requirement of DNA polymerase η for error-free bypass of UV-induced CC and TC photoproducts. Mol Cell Biol. 2001;21: 185–188. 11113193 10.1128/MCB.21.1.185-188.2001 PMC88792

[40] Budd ME, Campbell JL. DNA polymerases required for repair of UV-induced damage in *Saccharomyces cerevisiae* . Mol Cell Biol. 1995;15: 2173–2179. 7891712 10.1128/mcb.15.4.2173 PMC230445

[41] Unrau P, Wheatcroft R, Cox B, Olive T. The formation of pyrimidine dimers in the DNA of fungi and bacteria. Biochim Biophys Acta. 1973;312: 626–632. 4200353 10.1016/0005-2787(73)90065-8

[42] Guo D, Wu X, Rajpal DK, Taylor JS, Wang Z. Translesion synthesis by yeast DNA polymerase ζ from templates containing lesions of ultraviolet radiation and acetylaminofluorene. Nucleic Acids Res. 2001;29: 2875–2883. 11433034 10.1093/nar/29.13.2875 PMC55783

[43] Abdulovic AL, Jinks-Robertson S. The *in vivo* characterization of translesion synthesis across UV-induced lesions in *Saccharomyces cerevisiae*: insights into Pol ζ- and Pol η-dependent frameshift mutagenesis. Genetics. 2006;172: 1487–1498. 16387871 10.1534/genetics.105.052480 PMC1456278

[44] Nelson JR, Lawrence CW, Hinkle DC. Thymine-thymine dimer bypass by yeast DNA polymerase ζ. Science. 1996;272: 1646–1649. 8658138 10.1126/science.272.5268.1646

[45] Johnson RE, Washington MT, Haracska L, Prakash S, Prakash L. Eukaryotic polymerases ι and ζ act sequentially to bypass DNA lesions. Nature. 2000;406: 1015–1019. 10984059 10.1038/35023030

[46] Acharya N, Johnson RE, Prakash S, Prakash L. Complex formation with Rev1 enhances the proficiency of *Saccharomyces cerevisiae* DNA polymerase ζ for mismatch extension and for extension opposite from DNA lesions. Mol Cell Biol. 2006;26: 9555–9563. 17030609 10.1128/MCB.01671-06 PMC1698531

[47] Bresson A, Fuchs RP. Lesion bypass in yeast cells: Pol η participates in a multi-DNA polymerase process. EMBO J. 2002;21: 3881–3887. 12110599 10.1093/emboj/cdf363 PMC126109

[48] Yang Y, Sterling J, Storici F, Resnick MA, Gordenin DA. Hypermutability of damaged single-strand DNA formed at double-strand breaks and uncapped telomeres in yeast *Saccharomyces cerevisiae* . PLoS Genet. 2008;4: e1000264 10.1371/journal.pgen.1000264 19023402 PMC2577886

[49] Smith DJ, Whitehouse I. Intrinsic coupling of lagging-strand synthesis to chromatin assembly. Nature. 2012;483: 434–438. 10.1038/nature10895 22419157 PMC3490407

[50] Waisertreiger IS, Liston VG, Menezes MR, Kim HM, Lobachev KS, Stepchenkova EI, et al Modulation of mutagenesis in eukaryotes by DNA replication fork dynamics and quality of nucleotide pools. Environ Mol Mutagen. 2012;53: 699–724. 10.1002/em.21735 23055184 PMC3893020

[51] Greenfeder SA, Newlon CS. Replication forks pause at yeast centromeres. Mol Cell Biol. 1992;12: 4056–4066. 1508202 10.1128/mcb.12.9.4056-4066.1992 PMC360298

[52] Fachinetti D, Bermejo R, Cocito A, Minardi S, Katou Y, Kanoh Y, et al Replication termination at eukaryotic chromosomes is mediated by Top2 and occurs at genomic loci containing pausing elements. Mol Cell. 2010;39: 595–605. 10.1016/j.molcel.2010.07.024 20797631 PMC3041477

[53] McGuffee SR, Smith DJ, Whitehouse I. Quantitative, genome-wide analysis of eukaryotic replication initiation and termination. Mol Cell. 2013;50: 123–135. 10.1016/j.molcel.2013.03.004 23562327 PMC3628276

[54] Drake JW. A constant rate of spontaneous mutation in DNA-based microbes. Proc Natl Acad Sci USA. 1991;88: 7160–7164. 1831267 10.1073/pnas.88.16.7160 PMC52253

[55] Nik-Zainal S, Alexandrov LB, Wedge DC, Van Loo P, Greenman CD, Raine K, et al Mutational processes molding the genomes of 21 breast cancers. Cell. 2012;149: 979–993. 10.1016/j.cell.2012.04.024 22608084 PMC3414841

[56] Roberts SA, Sterling J, Thompson C, Harris S, Mav D, Shah R, et al Clustered mutations in yeast and in human cancers can arise from damaged long single-strand DNA regions. Mol Cell. 2012;46: 424–435. 10.1016/j.molcel.2012.03.030 22607975 PMC3361558

[57] Taylor BJ, Nik-Zainal S, Wu YL, Stebbings LA, Raine K, Campbell PJ, et al DNA deaminases induce break-associated mutation showers with implication of APOBEC3B and 3A in breast cancer kataegis. Elife. 2013;2: e00534 10.7554/eLife.00534 23599896 PMC3628087

[58] Camps M, Herman A, Loh E, Loeb LA. Genetic constraints on protein evolution. Crit Rev Biochem Mol Biol. 2007;42: 313–326. 17917869 10.1080/10409230701597642 PMC3825456

[59] Stone JE, Lujan SA, Kunkel TA, Kunkel TA. DNA polymerase ζ generates clustered mutations during bypass of endogenous DNA lesions in *Saccharomyces cerevisiae* . Environ Mol Mutagen. 2012;53: 777–786. 10.1002/em.21728 22965922 PMC3678557

[60] Drake JW, Bebenek A, Kissling GE, Peddada S. Clusters of mutations from transient hypermutability. Proc Natl Acad Sci USA. 2005;102: 12849–12854. 16118275 10.1073/pnas.0503009102 PMC1200270

[61] Crosby B, Prakash L, Davis H, Hinkle DC. Purification and characterization of a uracil-DNA glycosylase from the yeast *Saccharomyces cerevisiae* . Nucleic Acids Res. 1981;9: 5797–5809. 7031606 10.1093/nar/9.21.5797 PMC327561

[62] Sharma S, Shah NA, Joiner AM, Roberts KH, Canman CE. DNA polymerase ζ is a major determinant of resistance to platinum-based chemotherapeutic agents. Mol Pharmacol. 2012;81: 778–787. 10.1124/mol.111.076828 22387291 PMC3362893

[63] Doles J, Oliver TG, Cameron ER, Hsu G, Jacks T, Walker GC, et al Suppression of Rev3, the catalytic subunit of Polζ, sensitizes drug-resistant lung tumors to chemotherapy. Proc Natl Acad Sci USA. 2010;107: 20786–20791. 10.1073/pnas.1011409107 21068376 PMC2996428

[64] Okuda T, Lin X, Trang J, Howell SB. Suppression of hREV1 expression reduces the rate at which human ovarian carcinoma cells acquire resistance to cisplatin. Mol Pharmacol. 2005;67: 1852–1860. 15758147 10.1124/mol.104.010579

[65] Xie K, Doles J, Hemann MT, Walker GC. Error-prone translesion synthesis mediates acquired chemoresistance. Proc Natl Acad Sci USA. 2010;107: 20792–20797. 10.1073/pnas.1011412107 21068378 PMC2996453

[66] Shcherbakova PV, Noskov VN, Pshenichnov MR, Pavlov YI. Base analog 6-*N*-hydroxylaminopurine mutagenesis in the yeast *Saccharomyces cerevisiae* is controlled by replicative DNA polymerases. Mutat Res. 1996;369: 33–44. 8700180 10.1016/s0165-1218(96)90045-2

[67] Shcherbakova PV, Pavlov YI. 3'—>5' exonucleases of DNA polymerases ε and δ correct base analog induced DNA replication errors on opposite DNA strands in *Saccharomyces cerevisiae* . Genetics. 1996;142: 717–726. 8849882 10.1093/genetics/142.3.717 PMC1207013

[68] Gueldener U, Heinisch J, Koehler GJ, Voss D, Hegemann JH. A second set of *loxP* marker cassettes for Cre-mediated multiple gene knockouts in budding yeast. Nucleic Acids Res. 2002;30: e23 11884642 10.1093/nar/30.6.e23 PMC101367

[69] Guldener U, Heck S, Fielder T, Beinhauer J, Hegemann JH. A new efficient gene disruption cassette for repeated use in budding yeast. Nucleic Acids Res. 1996;24: 2519–2524. 8692690 10.1093/nar/24.13.2519 PMC145975

[70] Shcherbakova PV, Kunkel TA. Mutator phenotypes conferred by *MLH1* overexpression and by heterozygosity for *mlh1* mutations. Mol Cell Biol. 1999;19: 3177–3183. 10082584 10.1128/mcb.19.4.3177 PMC84111

[71] Shcherbakova PV, Pavlov YI. Mutagenic specificity of the base analog 6-*N*-hydroxylaminopurine in the *URA3* gene of the yeast *Saccharomyces cerevisiae* . Mutagenesis. 1993;8: 417–421. 8231822 10.1093/mutage/8.5.417

[72] Lada AG, Waisertreiger IS, Grabow CE, Prakash A, Borgstahl GE, Rogozin IB, et al Replication protein A (RPA) hampers the processive action of APOBEC3G cytosine deaminase on single-stranded DNA. PLoS One. 2011;6: e24848 10.1371/journal.pone.0024848 21935481 PMC3174200

[73] Sikorski RS, Hieter P. A system of shuttle vectors and yeast host strains designed for efficient manipulation of DNA in *Saccharomyces cerevisiae* . Genetics. 1989;122: 19–27. 2659436 10.1093/genetics/122.1.19 PMC1203683

[74] Banerjee SK, Borden A, Christensen RB, LeClerc JE, Lawrence CW. SOS-dependent replication past a single *trans-syn* T-T cyclobutane dimer gives a different mutation spectrum and increased error rate compared with replication past this lesion in uninduced cells. J Bacteriol. 1990;172: 2105–2112. 2180917 10.1128/jb.172.4.2105-2112.1990 PMC208710

[75] Wang Z, Rossman TG. Isolation of DNA fragments from agarose gel by centrifugation. Nucleic Acids Res. 1994;22: 2862–2863. 7519772 10.1093/nar/22.14.2862 PMC308261

[76] Amberg DC, Burke DJ, Strathern JN. High-efficiency transformation of yeast. CSH Protoc. 2006;2006. 10.1101/pdb.prot4145 22485546

[77] Northam MR, Robinson HA, Kochenova OV, Shcherbakova PV. Participation of DNA polymerase ζ in replication of undamaged DNA in *Saccharomyces cerevisiae* . Genetics. 2010;184: 27–42. 10.1534/genetics.109.107482 19841096 PMC2815923

[78] Drake JW, Charlesworth B, Charlesworth D, Crow JF. Rates of spontaneous mutation. Genetics. 1998;148: 1667–1686. 9560386 10.1093/genetics/148.4.1667 PMC1460098

