# Supplementary material for: DNA Polymerase ζ-Dependent Lesion Bypass in Saccharomyces cerevisiae Is Accompanied by Error-Prone Copying of Long Stretches of Adjacent DNA
Source: PLoS Genet. 2015 Mar 31;11(3):e1005110. doi: 10.1371/journal.pgen.1005110 (PMC4380420; doi:10.1371/journal.pgen.1005110)
Supplement: S2 Table — The location of the potential photolesion site and the true and pseudo reversion pathways are explained in detail in Fig. 3. (DOCX) [file pgen.1005110.s002.docx]

**S2 Table.** Nucleotide changes at the site of the presumed UV lesion at positions 763-765 of the *URA3* gene in UV-induced revertants in the *rad30Δ* background.

| **Reversion type** | **Number of**  **occurrences** |
| --- | --- |
| GAC → **A**AC | 45 |
| GAC → **T**AC | 28 |
| GAC → **C**AC | 2 |
| GAC → G**G**C | 42 |
| GAC → G**T**C | 19 |
| GAC → G**C**C | 26 |
| GAC → G**CT** | 1 |
| AGAC → **GT**AC | 1 |
| AGAC → **CC**AC | 1 |

The location of the potential photolesion site and the true and pseudo reversion pathways are explained in detail in Fig. 3.
